# Supplementary figures and images for: Winter diet and food selection of the Black-necked Crane Grus nigricollis in Dashanbao, Yunnan, China
Source: PeerJ. 2016 Apr 21;4:e1968. doi: 10.7717/peerj.1968 (PMC4846803; doi:10.7717/peerj.1968)

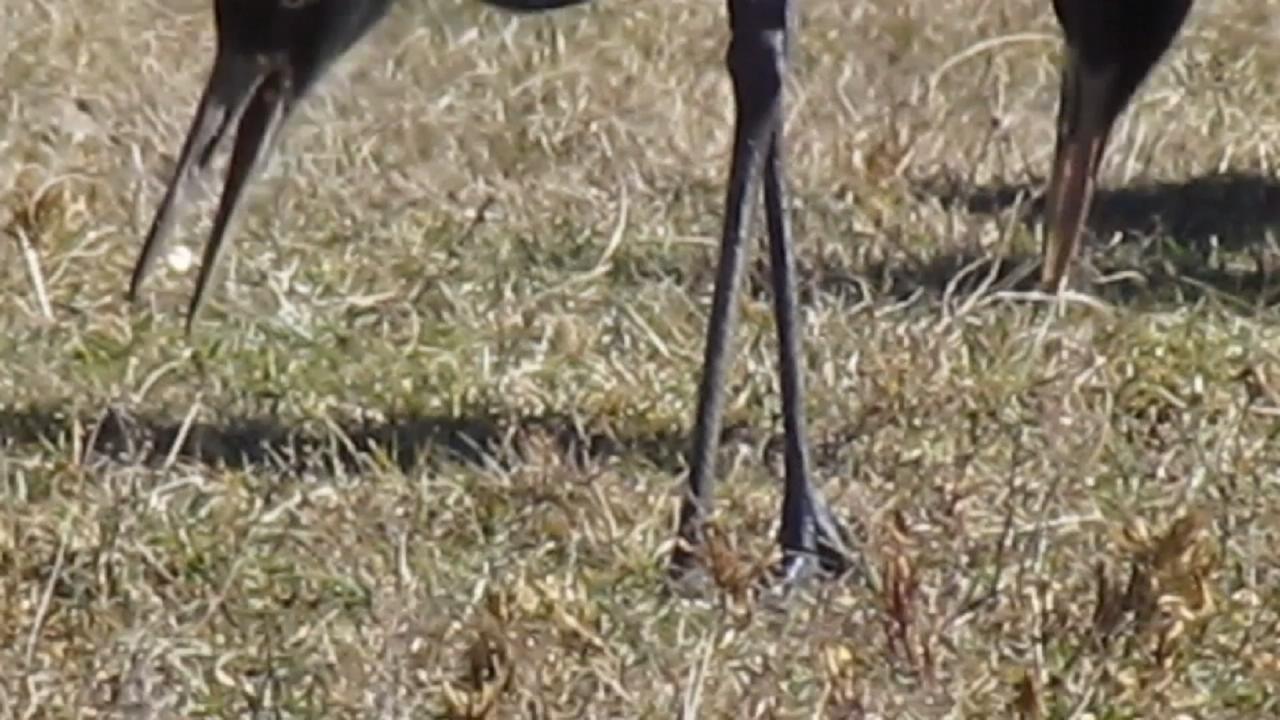

Supplement: Figure S1 [file peerj-04-1968-s001.jpg]

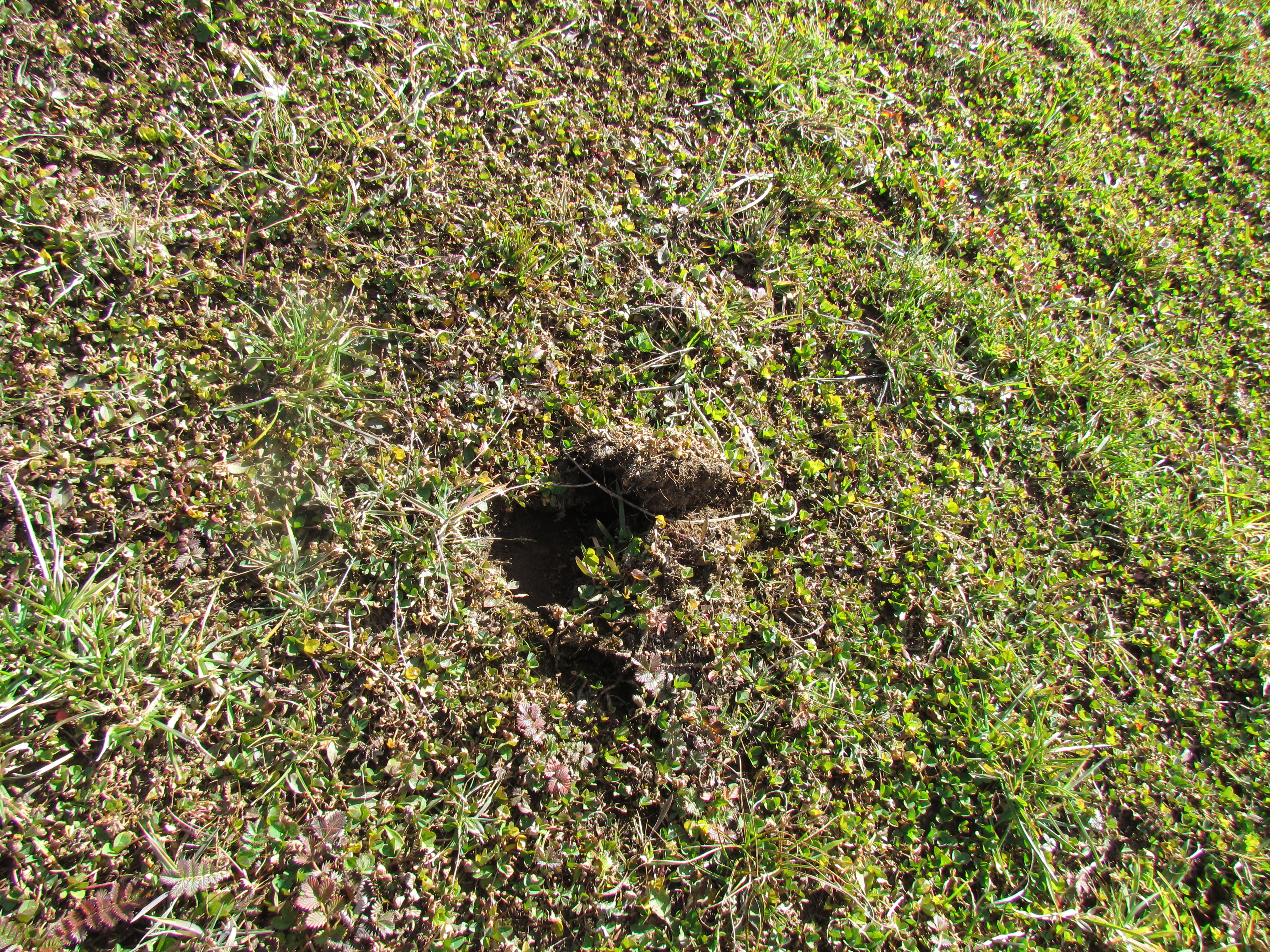

Supplement: Figure S2 [file peerj-04-1968-s002.jpg]

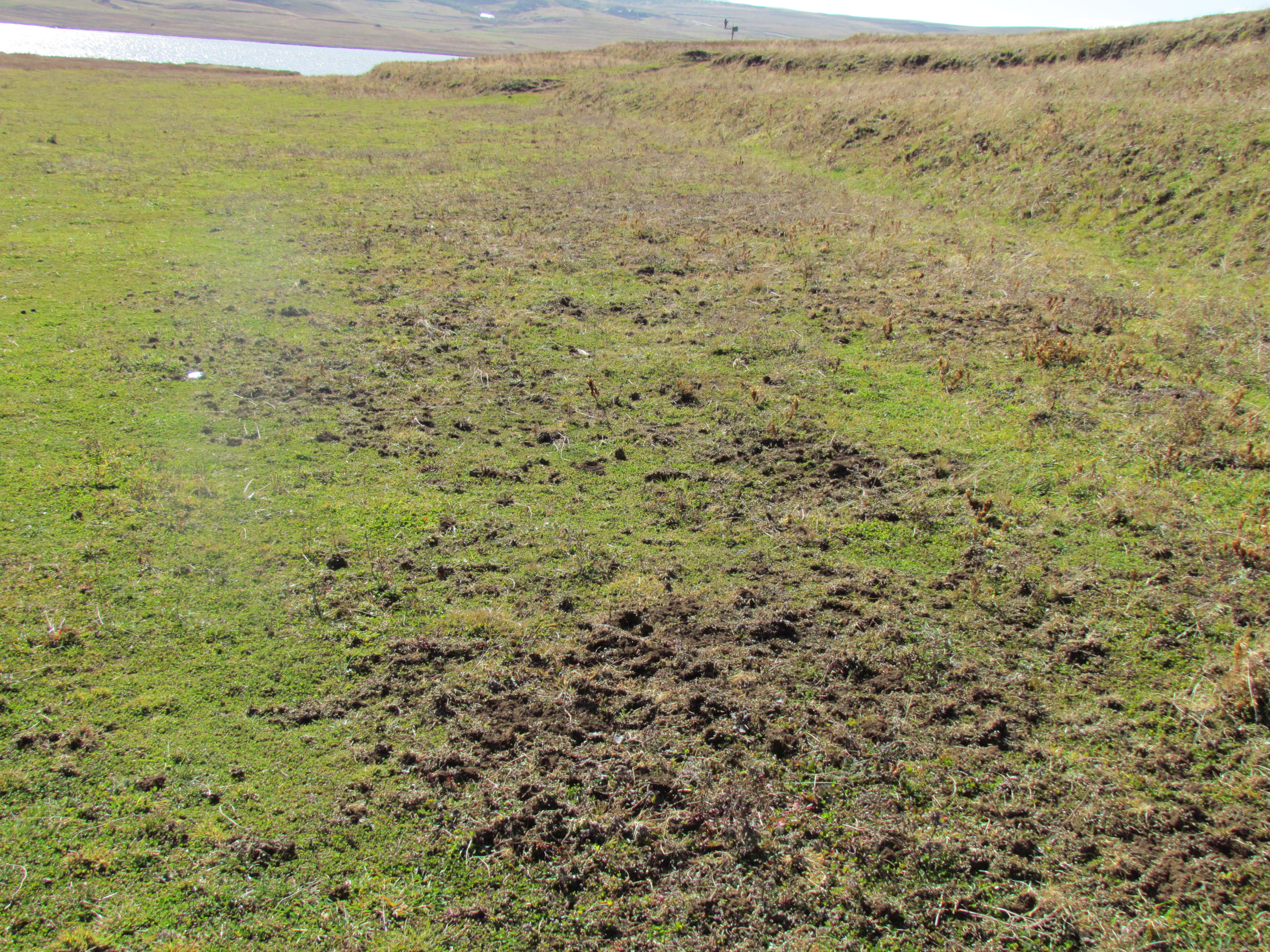

Supplement: Figure S3 [file peerj-04-1968-s003.jpg]

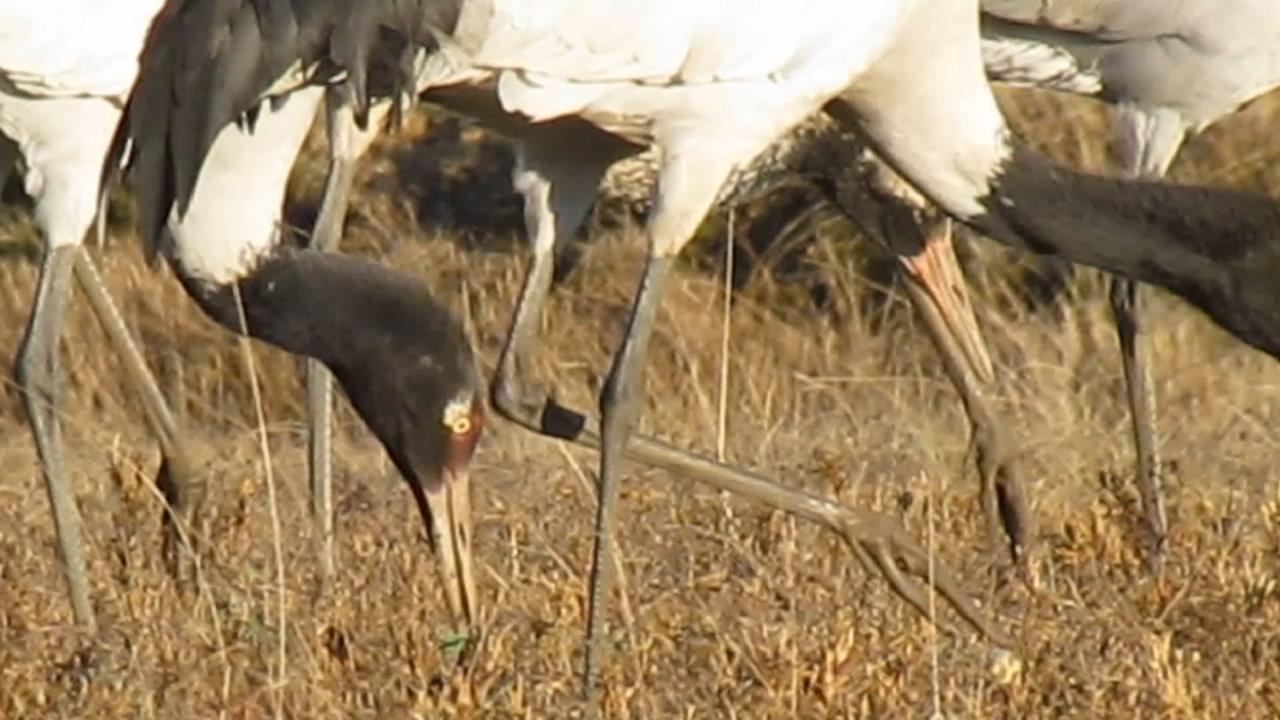

Supplement: Figure S4 [file peerj-04-1968-s004.jpg]

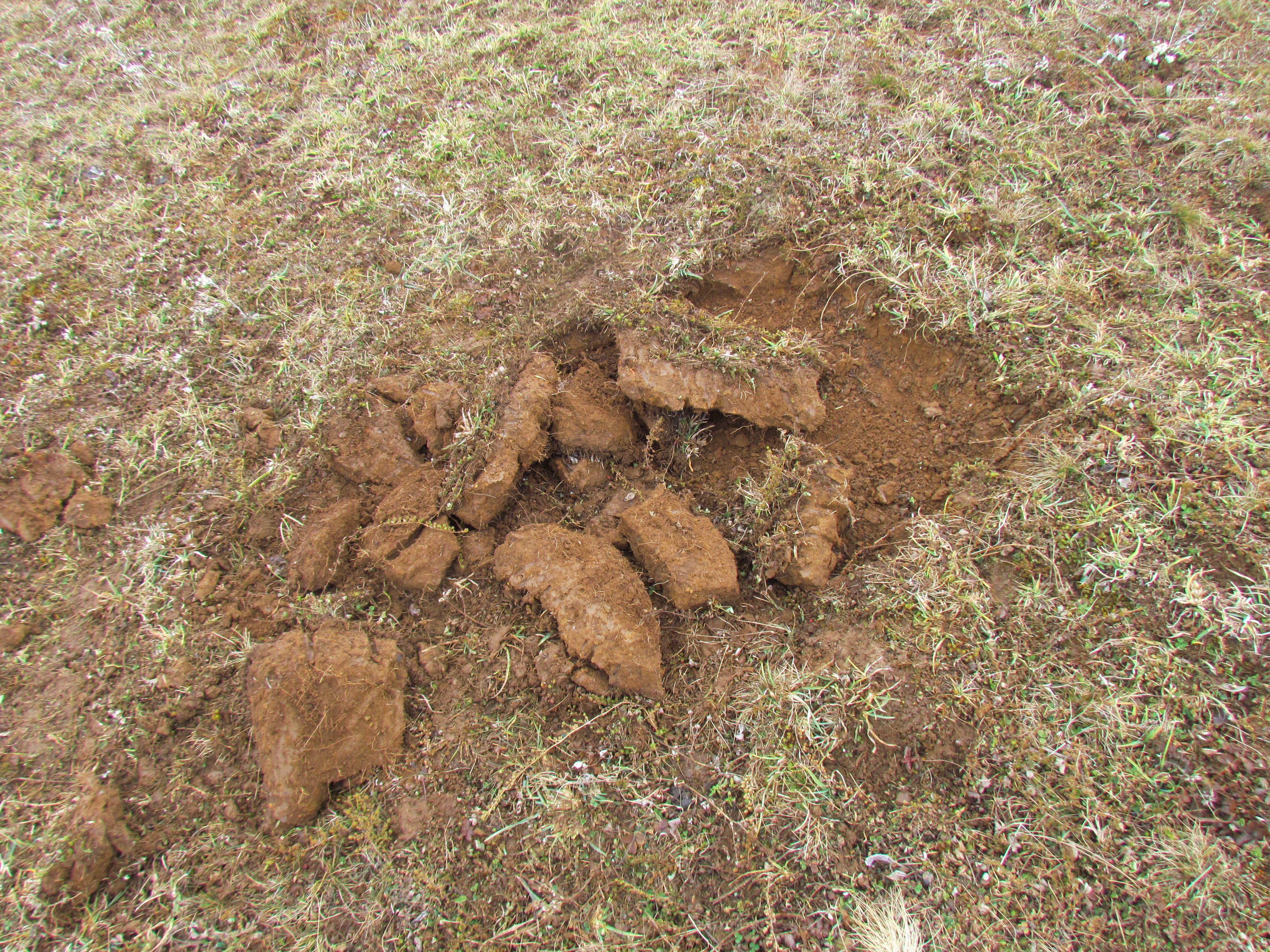

Supplement: Figure S5 [file peerj-04-1968-s005.jpg]
